# Impact of cancer evolution on immune surveillance and checkpoint inhibitor response

**DOI:** 10.1016/j.semcancer.2021.02.013

**Published:** 2022-09

**Authors:** Yin Wu, Dhruva Biswas, Charles Swanton

**Affiliations:** a Cancer Evolution and Genome Instability Laboratory, The Francis Crick Institute, London, NW1 1AT, UK; b Cancer Research UK Lung Cancer Centre of Excellence, University College London Cancer Institute, Paul O’Gorman Building, London, WC1E 6DD, UK; c Peter Gorer Department of Immunobiology, School of Immunology & Microbial Sciences, King’s College London, London, SE1 9RT, UK; d Bill Lyons Informatics Centre, University College London Cancer Institute, Paul O’Gorman Building, London, WC1E 6DD, UK

**Keywords:** Intratumour heterogeneity, Neoantigens, Genomic instability, Immune surveillance, Immune checkpoint inhibitors

## Abstract

Intratumour heterogeneity (ITH) is pervasive across all cancers studied and may provide the evolving tumour multiple routes to escape immune surveillance. Immune checkpoint inhibitors (CPIs) are rapidly becoming standard of care for many cancers. Here, we discuss recent work investigating the influence of ITH on patient response to immune checkpoint inhibitor (CPI) therapy. At its simplest, ITH may confound the diagnostic accuracy of predictive biomarkers used to stratify patients for CPI therapy. Furthermore, ITH is fuelled by mechanisms of genetic instability that can both engage immune surveillance and drive immune evasion. A greater appreciation of the interplay between ITH and the immune system may hold the key to increasing the proportion of patients experiencing durable responses from CPI therapy.

## 1 Introduction

The last decade has witnessed a profound change in our understanding and treatment of cancer. Decreasing costs of next generation sequencing (NGS) have aided the study of the genomic, epigenomic and transcriptomic landscape of tumours [[1], [2], [3], [4]]. This has helped the community understand the complexity of established tumours, for example through establishing a catalogue of “driver” genes that are causally implicated in tumourigenesis [5]. Moreover, it has supported the development of targeted therapies and companion diagnostics [[6], [7], [8], [9]] as well as the identification of mechanisms of therapeutic resistance [10,11]. Alongside advances in our understanding of cancer biology, the last decade has also witnessed advances in cancer immunotherapy, notably in the translation of preclinical immune checkpoint inhibitors [[12], [13], [14], [15]] to clinical standard of care [[16], [17], [18], [19]].

Within cancer biology, a major focus of recent study has been the burgeoning field of intratumour heterogeneity (ITH) and cancer evolution. It has long been proposed that tumorigenesis is a branching multistep, non-linear process, involving the selection of variant subclones within tumours [20,21]. Early studies demonstrated the existence of phenotypic ITH in characteristics such as histology and therapeutic sensitivity [[22], [23], [24], [25], [26], [27], [28]]. However, it was not until recently that the extent of genetic ITH was appreciated. Mutations such as somatic point mutations, copy number alterations and chromosomal level alterations drive cancer genomic instability leading to cell-to-cell variation and genetic ITH [[29], [30], [31], [32], [33], [34]]. It is now well established that most cancers undergo a Darwinian-like process of evolution. “Clonal” mutations are detectable in all cancer cells and so are thought to occur early in the life history of a tumour, forming the trunk of its phylogenetic tree. Most clinically actionable driver mutations tend to be clonal. By contrast, subsequent “subclonal” mutations are present in a fraction of cancer cells, forming the branches of the phylogenetic tree [[35], [36], [37], [38], [39], [40], [41]] and may confer a selective advantage within specific microenvironmental contexts [[42], [43], [44]]. Thus, ITH exists not only in the histopathological appearance of tumours and in differences of stromal infiltrates but also at the genetic level with co-existence of subclonal lineages within the same tumour. A tumour with a greater number and/or proportion of subclonal mutations is generally considered to have a high degree of genetic ITH. However, there is no consensus currently over which is more important, the absolute number of subclonal mutations or their relative proportion. Multi-region sampling paired with deep sequencing has now demonstrated the existence of this genetic ITH essentially in all cancers studied [40]. ITH in turn provides the substrate on which Darwinian selection acts and resistance to treatment evolves [45]. The overarching impact of ITH on prognosis has been demonstrated prospectively in NSCLC [33] and retrospectively in pan-cancer analyses where high levels of ITH are independently correlated with worse outcome [46,47].

The failure of targeted therapies to sustain long term disease control against dominant clonal driver mutations can be better appreciated in light of ITH. Pre-existing tumour cells harbouring resistance mutations, are eventually selected for under therapeutic pressure [[48], [49], [50], [51]]. However, resistance is not always a fait accompli as *de novo* mutations are also a route towards therapeutic resistance [[52], [53], [54], [55]]. For example, Russo and colleagues recently demonstrated that targeted therapy with EGFR or BRAF inhibition in colorectal cancer cell lines induced genetic instability leading to *de novo* mutagenesis through the downregulation of DNA repair machinery, potentially driving resistance [55].

The implications of ITH on immunotherapy are less well understood. Unlike targeted therapy, which is static, the immune system is a dynamic entity with the potential to keep pace with tumour evolution [56]. Here we review and discuss evidence around the impact of ITH on immune surveillance, focusing on both genetic and non-genetic (stromal) ITH and immune checkpoint inhibitors (CPIs). CPIs are now becoming standard of care for many cancers but remain effective only for a minority of patients. Moreover, they are costly and come with significant risk of lifelong toxicities [[57], [58], [59]]. Thus, a greater understanding of how ITH influences CPI therapy is critical to guide judicious use of these therapies.

## 2 Adaptive immune recognition of tumours and immunological checkpoints

**Fig. 1:**
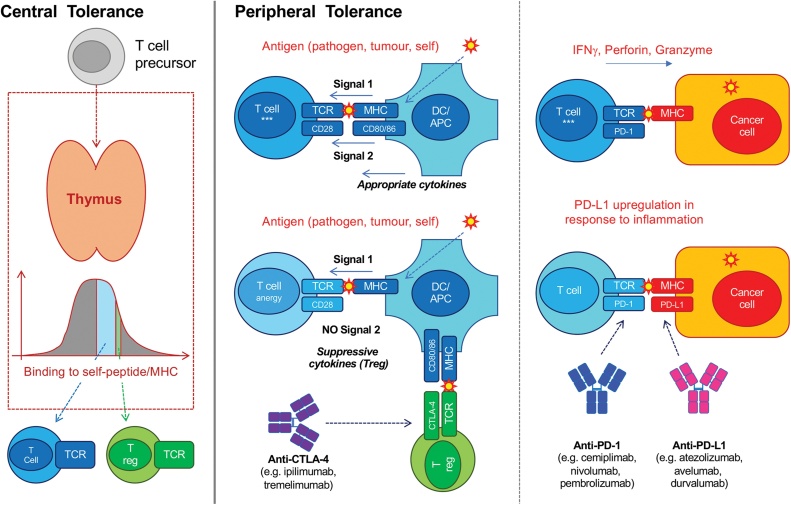
T cell development and activation is highly regulated to avoid autoimmunity. During thymic development, only thymocytes (T cell precursors) with moderate affinity to self-peptide-MHC progress to become mature naïve T cells. Thymocytes with high affinity for self-peptide-MHC die by apoptosis, whilst those with low affinity die by neglect. Thymocytes with moderately high affinity for self-peptide-MHC become regulatory T cells (Tregs) in the periphery. This process of “central tolerance” prevents some, but not all, autoreactive T cells from being released into the periphery. Despite central tolerance, many autoreactive mature naïve T cells are released from the thymus into the periphery. Thus, several physiological mechanisms exist in the periphery to mitigate autoimmunity as part of “peripheral tolerance”. Activation of mature naïve T cells in the periphery requires specific TCR signalling via cognate peptide presented on MHC (“Signal 1″) in addition to contemporaneous signalling via CD28 and co-stimulatory receptors (e.g. CD80/CD86) on professional antigen presenting cells (“Signal 2″). The combination of contemporaneous Signal 1 and Signal 2 thus licenses naïve T cells to become effector memory T cells. The local cytokine environment also potentiates activation and skews functional responses. Tumour antigens are often similar to self-antigens and thus may also engage and activate Tregs (enriched for self-reactivity). Tregs express high levels of the inhibitory co-receptor CTLA-4 which has a higher binding affinity for CD80 and CD86 than CD28. Thus, Tregs sequester co-stimulatory CD80/CD86 and deprive other T cells of Signal 2. In the absence of a co-stimulatory signal, mature naïve T cells are by default anergised instead of activated, whilst memory T cells are less effectively activated. Moreover, Tregs secrete immunosuppressive cytokines (e.g. IL-10) which suppress nearby T cells in a paracrine fashion. Anti-CTLA-4 CPIs bind to and block CTLA-4 sequestration of co-stimulation as well as deplete Tregs and thus favour T cell activation. Activated T cells can secrete inflammatory cytokines (e.g. IFNγ) or cytolytic effector molecules (e.g. perforin/granzyme) in response to target cells (e.g. cancer cells, pathogen infected cells). This in turn induces expression of inhibitory ligands, such as PD-L1, in target cells and nearby cells in a homeostatic fashion to mitigate excessive tissue damage. PD-L1 binding to PD-1 expressed on activated T cells attenuates TCR signalling. Anti-PD-(L)1 CPIs block this interaction and thus favour T cell activation.

T cells can be a potent defence against neoplasia [[60], [61], [62], [63], [64], [65], [66]] but they are also a major contributor to autoimmunity [[67], [68], [69], [70]]. Adaptive T cells use the αβ T cell receptor (TCR) which recognises cognate antigens via short peptide epitopes presented in the context of major histocompatibility (MHC) molecules (Fig. 1). Through a process of somatic recombination, a vast number (∼10^16^) of unique TCRs can be generated thus conferring αβ T cells with sufficient diversity to recognise essentially any antigen [[71], [72], [73]]. Given the stochastic nature by which this immense diversity is generated, the development and activation of αβ T cells is highly regulated to guard against detrimental recognition of self and autoimmunity (Fig. 1). αβ T cells that recognise self-peptides with high affinity are removed during “central” thymic development and silenced in the periphery [69]. Activated effector T cells are therefore enriched in non-self-reactive specificities. This has profound implications on the recognition of tumours by αβ T cells. Whilst αβ T cells may be exquisitely sensitive to bacterial or viral antigens, which are largely evolutionarily distinct from human self-antigens, their recognition of malignant cells is greatly hampered by similarity to self. Thus, the presentation of tumour specific antigens, such as neoantigens resulting from genomic aberrations including single nucleotide variants (SNV), frameshift mutations and/or insertion/deletion events (indels) [[74], [75], [76]] or ectopically expressed antigens not normally seen in healthy somatic tissues, such as cancer-testis antigens [77,78], is critical for tumour targeting by αβ T cells. Indeed, neoantigens which share homology with pathogen derived epitopes have recently been demonstrated to be more effective at engaging T cell immune surveillance [79]. In addition to antigenic visibility, αβ T cells also require contemporaneous signals from other immune cells, particularly innate immune cells. This both licenses αβ T cells to respond (Fig. 1) and shapes the quality of that response. Thus, for effective αβ T cell tumour immune surveillance, there is a requirement for an appropriate cellular and cytokine environment, in particular professional antigen presenting cells [80] and T-helper 1 (Th1) cytokines [[81], [82], [83], [84], [85]]. Whilst other T cells are found in tumours (e.g. γδ T cells and natural killer T cells) and also play an important role in cancer immune surveillance [61,65,[86], [87], [88], [89], [90], [91]], this review will focus on αβ T cells in the context of CPI therapy (henceforth referred to as T cells).

As part of a fail-safe peripheral tolerance programme to mitigate autoimmunity, multiple physiological immune checkpoints exist to restrain inappropriate and/or exuberant T cell activation [92]. These immune checkpoints are hard-wired and switched on as soon as T cells are activated, regardless of the stimuli and are not specific to cancer [93,94]. Current standard of care CPIs target one of two major immune checkpoints, specifically the maintenance of peripheral tolerance by the CTLA-4 axis and the restraint of excessive T cell activation in tissues, by the CTLA-4 and PD-1 axes. CPIs are monoclonal antibodies which bind to and disrupt signalling via these inhibitory receptors on T cells (Fig. 1) and thus favour T cell activation.

## 3 ITH and diagnostic challenges in the era of immune checkpoint inhibitors

De-repression of T cells by CPI therapy is important but not sufficient for effective tumour rejection. The efficacy of CPI therapy is maximised in the presence of appropriate tumour-associated antigens and immune milieu [[95], [96], [97], [98]]. Hence, tumours expressing higher levels of inhibitory immune checkpoint ligands (e.g. PD-L1) and those with higher tumour mutational burdens (TMBs), a surrogate for neoantigen burden, should be more likely to respond to CPI therapy. Indeed, the presence of PD-L1 does partially predict responses to anti-PD-(L)1 CPIs in several cancers including NSCLC, triple-negative breast, cervical, gastric/gastroesophageal and urothelial cancers [[97], [98], [99], [100], [101], [102]] and, in some cases, is used to guide anti-PD-(L)1 therapy [103]. Likewise, melanoma, non-small cell lung cancer (NSCLC) and DNA mismatch repair (MMR) deficient tumours, where CPIs have proven to be particularly effective, are amongst the cancers harbouring the highest TMBs [3,[104], [105], [106], [107], [108]]. Moreover, several studies have also demonstrated that a higher TMB or predicted neoantigen burden in pre-treatment biopsies predicts CPI response [[109], [110], [111], [112]]. Indeed, an assay for TMB has also been developed to estimate genome-wide mutation rates from the targeted sequencing of ∼3% of the coding genome in an effort to guide CPI therapy [113]. In the United States, the Food and Drug Administration has recently approved the use of the anti-PD-1 CPI, pembrolizumab, for the treatment of adult and paediatric patients with TMB-high (≥10 mutations/megabase) unresectable solid tumours who have no further standard of care treatment options. However, many patients derive no benefit from CPI therapy despite their tumours expressing high levels of PD-L1 and/or harbouring high predicted neoantigen burdens. Equally, many patients whose tumours express low levels of PD-L1 and/or harbour low predicted neoantigen burdens demonstrate robust responses to CPIs [[97], [98], [99],^109^,^110^,^114^].

ITH may impact CPI therapy response. At its simplest, ITH may give rise to the diagnostic challenge of tumour sampling bias. Several studies have demonstrated high rates of discordance in PD-L1 expression between multiple biopsies taken from the same tumour, and between diagnostic biopsies and surgically resected tumours [[115], [116], [117], [118], [119], [120]]. Similarly, the TRACERx consortium recently demonstrated discordant TMBs between biopsy sites from treatment-naïve primary NSCLC tumours in 21 % of patients when using the clinically relevant cutoff of TMB > 10 mutations/megabase [121]. This was independently confirmed in a subsequent study which reported a discordance rate of 30 % [122]. Sampling bias associated with ITH may contribute to why neither PD-L1 expression nor predicted neoantigen burden are perfect predictors of CPI response although this remains to be formally demonstrated. Multi-region sampling could potentially mitigate the effects of sampling bias but is not possible in routine care. “Liquid” biopsies provide a window on tumour somatic events through the molecular analysis of peripheral blood samples. This offers a pragmatic means to overcome sampling bias by assaying tumour material shed into the blood from multiple sites and has the added potential for longitudinal disease monitoring. For example, circulating tumour-derived DNA can be used to recapitulate clonal evolution of the primary tumours [123] or detect resistance mutations to targeted therapy in distinct subclones or different metastatic lesions [124]. Gandara and colleagues have recently demonstrated the utility of liquid biopsies for quantifying TMB in a retrospective analysis of two large clinical trials of atezolizumab (an anti-PD-L1 CPI) for patients with advanced NSCLC where they found good concordance between blood TMB (bTMB) and tumour TMB. Moreover, they showed that bTMB could be used to predict benefit from atezolizumab [125].

## 4 ITH and neoantigens

In addition to confounding the quantification of predictive biomarkers such as PD-L1 or predicted neoantigen burden, ITH itself may also directly impact the efficacy of CPI therapy. Neoantigens are required for visibility of tumours to the adaptive immune system. There is now mounting evidence that the quality, in addition to quantity, of neoantigens is critical for the immunosurveillance of cancers [126]. Like other mutations, neoantigens can be either clonal or subclonal. Clonal neoantigens are found in all tumour cells and form the trunk of the tumour phylogenetic tree whilst subclonal neoantigens are found in some but not all tumour cells and form the branches of the tumour phylogenetic tree [127]. Thus, tumours that harbour a greater number and/or proportion of subclonal neoantigens are considered to have higher ITH. From an ITH perspective, it appears that clonal neoantigens may be qualitatively superior than subclonal neoantigens in terms of engaging immune surveillance. Indeed, a recent pan-cancer analysis found that high ITH was associated with a lower levels of TILs [46]. McGranahan and colleagues were the first to demonstrate that the number of clonal neoantigens is both prognostic in early-stage tumours and a better predictor of CPI response than neoantigens alone in two cohorts of melanoma patients [127]. The prognostic impact of clonal neoantigen burden was confirmed in a recent study from the TRACERx consortium where a high absolute number of clonal neoantigens was associated with longer relapse-free survival in NSCLC in a multivariate model that included age, gender, histology, stage, pack years and adjuvant therapy. Conversely, subclonal neoantigens were not predictive of outcome in this analysis. This was in a cohort of patients with early-stage NSCLC who did not receive adjuvant CPI therapy before relapse, perhaps reflecting improved natural immune surveillance of clonal neoantigens [121]. Subsequent larger studies have since independently confirmed the predictive impact of clonal neoantigens in the context of CPI therapy [128,129]. A recent meta-analysis by Litchfield and colleagues of >1000 CPI-treated patients across 8 tumour-types suggests that the strongest predictor of response to CPI therapy was absolute clonal TMB [130]. Indeed, cancers such as Merkel cell cancer and Hodgkin lymphoma, which have a relatively low TMB but which also harbour clonal Merkel cell polyomavirus and Epstein-Barr virus genomes [131,132] demonstrate notably high response rates to CPI therapy [[133], [134], [135]]. Likewise, despite a relatively high TMB, melanomas with higher ITH, both in terms of proportion and number of subclonal mutations, have been reported to have lower response rates to CPI therapy [136].

As is often the case with correlative clinical data, it can be difficult to tease apart cause and effect. On the one hand, the association of clonal neoantigens with survival and CPI response would suggest that clonal neoantigens are superior to subclonal neoantigens in engaging tumour-rejecting T cells. Unlike clinical studies which are often correlative by necessity, murine studies enable controlled experimental manipulations to establish causation, albeit in a model of disease. Two recent studies using murine models support the hypothesis that clonal neoantigens are qualitatively superior at engaging effective tumour immune surveillance. In the first, Gejman and colleagues demonstrated that a minimum fraction of tumour cells must express a neoantigen before adaptive immune rejection and also that this dosage threshold varies for different neoantigens [137]. In the second study, Wolf and colleagues demonstrated, in a murine model of melanoma, that homogeneous, genetically clonal tumours were more readily rejected by the immune system compared with heterogenous tumours, independent of total mutational burden [136]. On the other hand, tumours undergo constant immune surveillance and thus clonal tumours may simply reflect a vigorous and efficient T cell rejection of tumour cells harbouring subclonal neoantigens. Along these lines, a recent computational pathology analysis of NSCLC patients enrolled in the TRACERx study discovered that tumours with high clonal neoantigen burdens were more densely infiltrated by T cells [138].

**Fig. 2:**
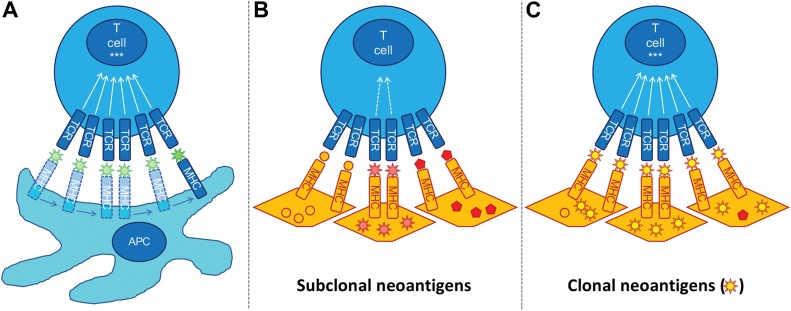
Clonal neoantigens may be superior in activating T cells compared with subclonal neoantigens. **(A)** Engagement of multiple TCRs is required for efficient T cell activation***. Cognate peptide-MHC complexes with optimal affinity (i.e. high enough to signal, low enough to dissociate and engage further TCRs) are able to serially engage TCRs for effective T cell signalling. **(B)** T cell activation is hindered by sub-optimal cognate peptide-MHC complex affinity. For example, high affinity interactions prolong TCR-peptide-MHC dwell time and thus impair serial engagement of TCRs. Subclonal neoantigens are especially handicapped by reduced serial TCR engagement due to relatively reduced peptide-MHC density. **(C)** Increased peptide-MHC density of clonal neoantigens (present on all cancer cells) can overcome limitations of non-optimal TCR affinity as there is less reliance on serial engagement of TCRs.

Why or if clonal neoantigens might present a higher quality antigen to the immune system is not certain. In the murine setting, there is evidence that neoantigen-directed T cell rejection of tumours requires a minimum clonal fraction and thus clonal neoantigens are more likely to induce T cell rejection [137]. Moreover, in some cancers such as NSCLC, whole genome doubling (WGD) occurs as an early clonal event [33,139]. Therefore, clonal mutations that occur prior to WGD have a higher gene-dosage and may be expressed at higher levels in tumour cells [140,141]. The nuanced requirements for optimal TCR signalling via peptide-MHC complexes may explain why clonal neoantigens appear to be more efficient at engaging immune surveillance. Effective T cell activation requires the serial engagement of its TCRs by cognate peptide-MHC complexes where the TCR occupancy is proportional to biological response [142]. To achieve serial engagement, TCR peptide-MHC interactions must be of a suitable affinity to both signal effectively and to dissociate readily, thus allowing relatively few peptide-MHC complexes to serially bind many TCRs [142,143]. Whilst there is a minimum affinity required for productive signalling at each TCR [[144], [145], [146]], high affinity interactions may impair overall T cell activation through prolonged dwell times and reduced serial TCR binding [147]. Increased peptide-MHC density has been shown to overcome the impairment of T cell activation by high affinity interactions as the abundance of peptide-MHC complexes compensates for reduced serial TCR engagement due to longer dwell times [148]. Thus, clonal neoantigens, by virtue of being expressed on all tumour cells, may provide the requisite peptide-MHC density to engage more TCRs than subclonal neoantigens, potentially overcoming suboptimal binding affinity (Fig. 2).

Whilst clonal neoantigens are likely to be higher quality overall, there is some evidence that targeting sub clonal neoantigens may have some merit in adoptive T cell therapy. A recent case report of successful autologous T cell therapy in metastatic breast cancer demonstrated that 2 out of 4 identified neoantigen-reactive T cell clones infused were directed against subclonal neoantigens [149]. However, it is worth noting that neoantigens may become pseudo-clonal at metastatic sites due to selection and bottlenecking. Moreover, in the context of the endogenous immune surveillance on which current CPI therapies work, there is strong evidence that a high proportion of subclonal neoantigens and a low proportion of clonal neoantigens is detrimental. Wolf and colleagues elegantly demonstrated this recently in a mouse model of melanoma [136]. They exposed the mouse melanoma B2905 cell line to UVB thereby inducing a greater proportion of subclonal mutations in UVB treated cells compared to the parental line. UVB treated cells grew markedly better in immunocompetent mice compared to the parental line whilst both grew equally well in NOD scid gamma (NSG) immunocompromised mice. The authors generated single cell clones (SCCs) from UVB irradiated B2905 cells and then systemically mixed SSCs to generate tumours with controlled proportions of subclonal versus clonal mutations. They demonstrated that tumours with a high proportion of subclonal versus clonal mutations were associated with a more immunosuppressive tumour microenvironment and less readily rejected in immunocompetent mice. Moreover, they demonstrated this was not because of an outgrowth of a resistant subclone suggesting that heterogeneity itself has an intrinsic impact on immune surveillance. Indeed, the authors concluded that high ITH actively impairs immune surveillance.

## 5 Interplay of genetic ITH, genomic instability and cancer immunity

ITH may reflect both neutral evolution and Darwinian selection of genetically diverse cancer cells due to stromal microenvironmental pressures present within tumours and the underlying genomic instability exhibited by the cancer cells themselves [150,151]. The impact of genomic instability on T cell immunosurveillance and response to CPI therapy is complex. On the one hand, genomic instability is important for the generation of somatic mutations and consequently neoantigens [[152], [153], [154], [155]]. Genomic instability is also intimately linked with activation of the innate immune system. For example, chromosomal instability (CIN), a form of genomic instability characterised by changes in the number and/or structure of chromosomes, is a major driver of micronuclei and cytosolic DNA in cancer cells [156]. The presence of cytosolic DNA activates the cGAS-STING pathway which in turn can induce expression of innate type 1 interferons [157,158]. Morever, cGAS-STING activation also induces expression of a major family of stress ligands for NKG2D, an activatory co-receptor found on innate and cytotoxic lymphocytes [159]. Induction of type 1 interferons and immunological stress ligands may provide the appropriate immune milieu to support tumour rejection by T cells, for example by increasing expression of MHC and co-stimulation on cancer cells. These data suggest that genomic instability could provide the optimal background for CPI therapy to act effectively.

Genomic instability directly affects not just the quantity but also the quality of neoantigens. Central and peripheral tolerance programmes (Fig. 1) select for T cells with low affinity for self-peptides. Hence, the more divergent tumour antigens are from self-antigens, the greater the likelihood that cognate T cells exist. Indeed, cancers which harbour non-self, viral antigens (e.g. Kaposi sarcoma, Merkel cell carcinoma, B-cell lymphomas) appear particularly sensitive to immune surveillance [160,161] and are often very responsive to CPI therapy [[133], [134], [135],^162^,^163^]. Beyond viral antigens, genomic instability can also contribute to divergence of tumour antigens from self-antigens [153,164]. For this reason, genomic instability may also be expected to associate with responsiveness to CPI therapy. There is certainly evidence for this in microsatellite instability high (MSI-H) tumours. These tumours have genomic instability due to deficiencies in the DNA MMR pathway [165,166] and accumulate large numbers of SNVs and indel mutations. Both SNVs and indel mutations can lead to neoantigens divergent from self. However, indel mutations, through frameshifts and generation of novel open reading frames, can generate more predicted neoantigens per mutation. Moreover, predicted neoantigens resulting from indel mutations are qualitatively different with a higher proportion predicted to be strong MHC binders [154]. MMR-deficient cancers show notably high responses to CPI therapy [106,107,167] and this appears to be largely driven by the burden of indel mutations [168]. Germano and colleagues recently demonstrated that inactivation of the mismatch repair gene MutL homologue 1 (MLH1) in murine colorectal cancer cell lines increased the susceptibility of transplanted tumours derived from these cells to immune surveillance and CPI therapy compared with tumours derived from parental cells [153]. Whilst these findings are at odds with those of Wolf and colleagues who used UVB to induce mutations and ITH, the two models are not directly comparable as MLH1 deficiency is more likely to generate indel mutations. Moreover, transplantable tumour models may be susceptible to evolutionary bottlenecking after transplantation, thereby forcing subclonal mutations to become clonal and thus more susceptible to immune surveillance (Fig. 2). Indeed, the authors report that subcloned MLH1-inactivated cells, which have a higher proportion of clonal neoantigens, were more readily rejected than non-subcloned MLH1-inactivated cells.

Although genomic instability may increase the quality of neoantigens and thus the potential for CPI responsiveness, it may also provide the necessary diversity from which therapy resistance and immune evasion evolve. Indeed CIN, is both a driver of intratumour heterogeneity and also linked to impaired immune surveillance [169] as well as resistance to CPI therapies [[170], [171], [172], [173]]. Mechanistically, CIN-high tumours may escape immune surveillance through chromosome level loss of genes or pathways implicated in effective surveillance. For example, type 1 interferons (e.g. interferon-α and interferon-β, IFN-α/β), are autocrine and paracrine cytokines that can induce senescence in stressed/transformed cells. Type 1 interferons can also potentiate tumour-rejecting immune responses including activating cytotoxic lymphocytes and upregulating the expression of MHC molecules and thus neoantigen visibility [174]. The type 1 interferon gene cluster has been reported to be lost in multiple cancers and its loss is associated with reduced immune cell infiltration and poor outcomes [[175], [176], [177], [178]]. This is consistent with recent work in a murine model which demonstrated CIN high tumours were more aggressive despite robust cGAS-STING activation. The authors showed this was associated with suppression of downstream type 1 interferon signalling and an increase in non-canonical NF-κB activation [156]. Loss of heterozygosity at the human leukocyte antigen locus (HLA, human MHC) is another phenomenon often found in cancers as a result of CIN and associated with high ITH. The loss of either the paternal or maternal alleles at the HLA locus reduces the breadth of tumour neoantigens that can be presented and thus limiting T cell visibility of tumour associated antigens [170]. This loss of T cell visibility would be expected to reduce the efficacy of CPI therapy. Consistent with this, it was recently demonstrated in NSCLC that patients who had high tumour mutational burdens (TMB) but also loss of heterozygosity at the HLA locus (LOHHLA) had inferior response rates to anti-PD-(L)1 therapy. The impact of LOHHLA in this cohort was notable as patients with high TMB but also LOHHLA had response rates equivalent to patients with low TMB [179]. Moreover, CIN might attenuate immune responses through global copy-number losses resulting in the deletion of clonal neoantigens [121]. Indeed, CIN has recently been implicated in acquired resistance to CPI therapy. Anagnostou and colleagues sequenced NSCLC tumours prior to CPI therapy and on progression post CPI therapy and demonstrated a chromosome level deletion of clonal neoantigens associated with an antigen specific T cell response in post progression tumours [180]. Whilst generally associated with resistance, copy-number events have also been linked with sensitisation to CPI therapy. A recent genome wide CRISPR screen identified TRAF2 as a key gene sensitising tumour cells to killing by T cell-derived tumour necrosis factor [181]. Consistent with this, TRAF2 loss was found in recent a pan-cancer analysis to be significantly associated with response to CPI therapy [130].

Immune activation, such as the production of type 1 interferons and expression of immunological stress ligands induced by genomic instability, can be a double-edged sword. Whilst acute immune activation is generally host protective, chronic activation has been associated with tumour progression, tolerance and resistance to CPI therapy [[182], [183], [184], [185], [186], [187]]. Unlike murine cancers which often follow a short evolutionary time course, most clinically apparent human cancers develop over years to decades and have a much greater burden of cells and genetic diversity [41].

## 6 Stromal ITH and CPI efficacy

Unsurprisingly, the efficacy of tumour immune surveillance is dependent not only on tumour cells, but also on stromal cells, in particular the immune microenvironment. Indeed, it is now well established that the presence or absence of tumour infiltrating leukocytes (TILs) predicts outcome in most cancers studied [188,189]. Whilst tumour cells largely dictate the neoantigen repertoire visible to T cells, the tumour immune stroma is largely responsible for providing the appropriate immune microenvironment for T cells to reject cancers. Moreover, many of the immunological checkpoints targeted by CPI therapy are also expressed on immune cells in addition to tumour cells [97]. Thus, it follows that the efficacy of CPI therapy is also partly dependent on the immune stroma. Indeed, the pre-treatment presence of TILs, in particular CD8 + T cells, has been shown to predict responses to CPI therapy [97,190], leading to suggestions that integrating TIL density with PD-L1 expression could refine the prediction of CPI sensitivity [191,192]. In addition to quantity, the quality of these TILs in terms of their functional differentiation [193] and antigen receptor repertoire have also been demonstrated to be important [194].

Like tumour cells themselves, the immune stroma also displays considerable intratumour heterogeneity, both quantitatively and qualitatively. Immune ITH has now been demonstrated in a myriad of cancers including lung, gastrointestinal, pancreatic, renal, ovarian and breast [121,138,[195], [196], [197], [198], [199], [200], [201], [202], [203], [204]]. Similar to predicted neoantigen burden and PD-L1 expression, the composition of the tumour immune stroma has been proposed as a predictive biomarker for CPI response [191,192,205,206]. Given the pervasiveness of immune stromal ITH, such biomarkers are likely to suffer from the same sampling biases. For example, histopathological analysis of metastatic samples from two large cohorts of colorectal patients revealed variable intra-patient TIL densities, and that the least immune-infiltrated biopsy was the strongest predictor of survival outcomes [207]. Likewise, a recent analysis, conducted in two large cohorts of NSCLC patients, revealed that lung tumours with at least two “immune-cold” biopsy sites had the poorest survival outcomes [138]. Whether the presence or degree of immune stromal ITH predicts response to CPI therapy is less certain. Immune stromal ITH itself is associated with tumour genetic ITH although it remains unclear to what degree the tumour shapes the immune stroma and/or vice versa [121,138,201,208].

## 7 Temporal ITH

Patients diagnosed with cancer are now surviving for longer [209,210]. Some patients are clear of macroscopic disease for many years before relapsing whilst others live with advanced cancer, often through many cycles of systemic anti-cancer therapy. Unsurprisingly, cancers at relapse or after several courses of therapy are often very different from the original primary. This temporal ITH is likely a result of selection pressures exerted by immune surveillance [136] and/or by anti-cancer therapies on pre-existing spatial ITH [30,48,211], as well as by different selection pressures at new sites of disease [212], or through acquired *de novo* mutations [[52], [53], [54], [55]]. Moreover, some therapies, particularly alkylating chemotherapies such as temozolomide and dacarbazine, may themselves induce *de novo* mutations through selection for MMR deficiency [37,127,213,214]. Temporal ITH adds a further layer of complexity. Biomarkers such as PD-L1 expression or TMB/predicted neoantigen burden derived from baseline biopsies or primary resections may be discordant with subsequent disease, particularly after systemic treatment [[215], [216], [217]], although it remains unclear if contemporaneous biopsies add further predictive value. An underappreciated but potentially concerning aspect of temporal ITH is the influence of accumulated *de novo* mutations induced by anti-cancer therapy. McGranahan and colleagues found that mutations associated with prior alkylating chemotherapy accounted for the majority of subclonal neoantigens in two patients with melanoma previously treated with dacarbazine, neither of whom responded to subsequent CPI therapy [127]. Acquired mutations, particularly in the DNA mismatch repair gene MSH6, are common in gliomas after treatment with temozolomide [213,214]. Although these tumours are deficient in DNA MMR and harbour high mutational burdens, recent single-centre studies suggest post-temozolamide MSH6 mutated hypermutant gliomas are poorly responsive to CPI therapy [218,219]. Thus, sequencing of CPI therapy in relation to lines of mutagenic chemotherapy may need to be considered to maximize the chances of response. This may be particularly relevant in cancers such as high-grade gliomas where most patients invariably receive first-line therapy with temozolomide [220] and where CPI therapy remains unproven [221,222]. Indeed, clinical trials are now underway to assess the efficacy of CPI therapy in the setting of relapsed gliomas where prior temozolomide therapy may obfuscate true efficacy of CPI therapy in this disease where few other efficacious treatments exist. Radiotherapy could also impact on ITH and response to CPI therapy. On the one hand, radiation can clearly alter the immune stroma of tumours and may synergise with immune surveillance to effect tumour control outside of the field of radiation [[223], [224], [225]]. On the other hand, radiotherapy can induce DNA damage, potentially leading to accumulation of subclonal *de novo* mutations and genetic ITH with implications on CPI response. There is currently little formal evidence in the literature of radiotherapy induced genetic ITH. However, a recent phase II trial of nivolumab in triple-negative breast cancer demonstrated a non-significant but numerically inferior response rate in patients who received induction radiotherapy compared with those that did not [226].

## 8 Concluding remarks

Immune checkpoint inhibitors are already now standard of care for five of the ten most common cancers in the UK (breast, lung, melanoma, renal, bladder). Meanwhile, ITH is virtually a hallmark of cancer and its implications for clinical practice are yet to be fully realised. This review has highlighted some of the recent work surrounding the impact of ITH on CPI therapy.

CPI therapies are expensive, toxic and benefit only a minority of cancer patients. Thus, many CPIs are only licensed for use alongside a companion predictive biomarker. For example, in the United Kingdom, the National Health Service funding of several anti-PD-(L)1 CPIs is predicated on a minimum expression of intratumoural PD-L1 (Table 1). Whilst these thresholds are useful, they are far from perfect in their utility to stratify patients. For patients with irresectable or metastatic disease, obtaining material for diagnostic assays commonly involves single site biopsies. Likewise, patients with relapsed disease do not always have contemporaneous biopsies. Thus, in routine clinical practice, spatial and temporal ITH may, in theory, impact on diagnostic accuracy. However, the degree of ITH in TMB when using the clinically relevant threshold of ≥10 mutations/megabase remains unclear [121,122,215,217]. Computational approaches to call clonal TMB from single samples [127] may mitigate sampling bias as clonal TMBs are by definition robust to ITH. Liquid biopsies may also help to overcome ITH. Liquid biopsies have already been demonstrated to overcome temporal heterogeneity in EGFR T790 M mutant NSCLC after treatment with EGFR inhibitors [[227], [228], [229]]. Liquid biopsies have also been demonstrated to detect spatial ITH in paired tumours [230] as well as to track clonal and subclonal metastatic relapse [123] and response to CPI therapy [231], albeit with limitations on sensitivity imposed by current technology.

**Table 1:** NHS funded CPI therapies requiring a threshold PD-L1 biomarker expression. (TILs) Tumour infiltrating leukocytes. (TPS) Tumour proportion score, the percentage of viable tumour cells showing partial or complete membrane staining at any intensity. (CPS) Combined positive score, the percentage of tumour cells and mononuclear inflammatory cells within tumour nests and adjacent supporting stroma expressing PD-L1 at any intensity. Adapted from NHS Cancer Drugs Fund List ver1.168 (20-Aug-2020) excluding interim additions for COVID19 pandemic.

CPI	Target	Indictation	PD-L1 Cutoff
Atezolizumab	PD-L1	First-line treatment of locally advanced/metastatic urothelial cancer	≥5% TILs
Atezolizumab	PD-L1	First-line treatment (in combination with bevacizumab, carboplatin and paclitaxel) of locally advanced or metastatic non-squamous NSCLC without activating EGFR/ALK mutation/ROS1 mutation	0−49% TPS
Durvalumab	PD-L1	Adjuvant treatment of locally advanced unresectable NSCLC following chemoradiation	≥1% TPS
Nivolumab	PD-1	Second-line(+) treatment of locally advanced/metastatic non-squamous NSCLC	≥1% TPS
Pembrolizumab	PD-1	First-line treatment of locally advanced/metastatic urothelial cancer	≥10 % CPS
Pembrolizumab	PD-1	Second-line treatment(+) of NSCLC without activating EGFR/ALK/ROS1 mutation	≥1% TPS
Pembrolizumab	PD-1	First-line treatment of NSCLC without activating EGFR/ALK/ROS1 mutation	≥50 % TPS

ITH itself appears to have direct implications on immune surveillance and response to checkpoint inhibitors. In general, ITH appears to be detrimental. ITH is associated with decreased immune surveillance, including decreased TILs and interferon signalling, across multiple cancers [232]. Murine models using tumours with controlled levels of ITH clearly demonstrate that increasing heterogeneity impairs immune surveillance and that this impairment is a function of the heterogeneity itself as opposed to outgrowth of resistant subclones [136]. Intriguingly, there may be an optimal threshold for evolvability, beyond which high ITH may tend towards genomic catastrophe [151] or potentially increased immune visibility [153]. In a meta-analysis of >2000 tumours, Birkbak and colleagues observed that patients with tumours that were in the highest quartile for CIN had improved survival compared to patients with tumours in the second-highest quartile who had the worst outcomes [233]. A subsequent pan-cancer analysis of >1000 tumours found that whilst tumours with the lowest ITH associated with the most favourable outcome, tumours with high ITH above a certain threshold also trended towards association with improved outcome [47]. Indeed, the definition of high ITH is not always clear in the literature. This term is often used interchangeably to mean either a high absolute subclonal mutational burden and/or a high proportion of subclonal mutations. Melanoma and NSCLC, where CPI therapies have demonstrated considerable efficacy, both have relatively high subclonal mutational burdens but also a relatively low proportion of subclonal mutations. Conversely, primary central nervous system cancers, where CPI therapies are generally less effective have a similar absolute number of subclonal mutations but these occupy a much greater proportion of total mutations [40]. Thus, we stress the urgent need for a clear and universal definition of ITH. Our growing recognition of ITH and the interplay with immune surveillance is beginning to shed light on our understanding of resistance to CPI therapy and may ultimately inform the development of more efficacious treatments.

## Declaration of Competing Interest

Y.W declares no conflicts of interest. D.B. acknowledges personal (speaking) fees from NanoString. D.B. has a patent on methods of predicting survival rates for patients with cancer pending. C.S. acknowledges grant support from Pfizer, AstraZeneca, Bristol Myers Squibb, Roche-Ventana, Boehringer-Ingelheim, Archer Dx Inc (collaboration in minimal residual disease sequencing technologies) and Ono Pharmaceutical, is an AstraZeneca Advisory Board member and Chief Investigator for the MeRmaiD1 clinical trial, has consulted for Pfizer, Novartis, GlaxoSmithKline, MSD, Bristol Myers Squibb, Celgene, AstraZeneca, Illumina, Genentech, Roche-Ventana, GRAIL, Medicxi and the Sarah Cannon Research Institute, has stock options in Apogen Biotechnologies, Epic Bioscience, GRAIL, and has stock options and is co-founder of Achilles Therapeutics. C.S. holds European patents relating to assay technology to detect tumour recurrence (PCT/GB2017/053,289); to targeting neoantigens (PCT/EP2016/059,401), identifying patent response to immune checkpoint blockade (PCT/EP2016/071,471), determining HLA LOH (PCT/GB2018/052,004), predicting survival rates of patients with cancer (PCT/GB2020/050,221), identifying patients who respond to cancer treatment (PCT/GB2018/051,912), a US patent relating to detecting tumour mutations (PCT/US2017/28,013) and both a European and US patent related to identifying insertion/deletion mutation targets (PCT/GB2018/051,892).
